# Time-resolving the ultrafast H_2_ roaming chemistry and H_3_^+^ formation using extreme-ultraviolet pulses

**DOI:** 10.1038/s42004-020-0294-1

**Published:** 2020-04-21

**Authors:** Ester Livshits, Itamar Luzon, Krishnendu Gope, Roi Baer, Daniel Strasser

**Affiliations:** 1 Institute of Chemistry, The Hebrew University of Jerusalem, Jerusalem, 91904 Israel; 2 Fritz Haber Center for Molecular Dynamics and the Institute of Chemistry, The Hebrew University of Jerusalem, Jerusalem, 91904 Israel

**Keywords:** Reaction kinetics and dynamics, Excited states

## Abstract

The time scales and formation mechanisms of tri-hydrogen cation products in organic molecule ionization processes are poorly understood, despite their cardinal role in the chemistry of the interstellar medium and in other chemical systems. Using an ultrafast extreme-ultraviolet pump and time-resolved near-IR probe, combined with high-level ab initio molecular dynamics calculations, here we report unambiguously that H_3_^+^ formation in double-ionization of methanol occurs on a sub 100 fs time scale, settling previous conflicting findings of strong-field Coulomb explosion experiments. Our combined experimental–computational studies suggest that ultrafast competition, between proton-transfer and long-range electron-transfer processes, determines whether the roaming neutral H_2_ dynamics on the dication result in ${\mathrm{H}}_3^ +$ or ${\mathrm{H}}_2^ +$ fragments respectively.

## Abstract (web summary)

The mechanism of formation of H_3_^+^ cations during double ionisation of methanol is debated. Here a combination of spectroscopic and molecular dynamics studies support a mechanism involving abstraction of a proton from the double cation by roaming neutral hydrogen molecules within 100 femtoseconds.

## Introduction

Trihydrogen ($${\mathrm{H}}_3^ +$$) is one of the most abundant ions in the universe with an important role in the formation of complex molecules in the interstellar medium (ISM)^1^. The formation of H_3_^+^ and particularly its destruction in the ISM attracts a vast experimental and theoretical effort^1–10^. While H_3_^+^ is typically formed by H_2_^+^ + H_2_ collisions^11^, it is a ubiquitous product of organic-molecule ionization processes such as electron impact^12^, fast ion bombardment^13^, multi-photon strong-field laser ionization^14,15^, as well as single extreme-ultraviolet (EUV) photon ionization^16^. However, the mechanisms for $${\mathrm{H}}_3^ +$$formation, as well as of the related $${\mathrm{H}}_2^ +$$ product, are still poorly understood. Early fragment imaging experiments using strong-field laser ionization by Yamanouchi and co-workers, provided first evidence as to the time scale of the $${\mathrm{H}}_3^ +$$ formation in ionization of methanol^14,15,17^. In contrast to products such as $$H^ +$$ and $${\mathrm{H}}_2^ +$$, in which the ejection directions exhibit a typical strong correlation with the strong-field laser polarization, $${\mathrm{H}}_3^ +$$ ejection from ionization of methanol was observed to be isotropic, suggesting rotational depolarization due to a “much greater” than 1.4 ps lifetime of the parent methanol dication^17^. A particularly long-time scale is in accord with the significant structural rearrangement required for H_3_^+^ formation from the parent methanol molecule. Early theoretical studies proposed an intricate mechanism involving formation of a neutral $${\mathrm{H}}_2$$ that roams on the doubly-ionized ground state potential surface of the organic molecule, culminating in proton abstraction and formation of $${\mathrm{H}}_3^ +$$^18,19^ The analogous roaming H-atom dynamics are known to exhibit prolonged roaming lifetimes ranging from hundreds of femtoseconds to thousands of picoseconds^20,21^.

Interpretation of multi-photon strong-field laser ionization experiments poses intrinsic challenges due to co-existence of direct and indirect ionization mechanisms. This intrinsic ambiguity has previously been the source of surprising and controversial results—for example concerning the double-proton transfer in a DNA base-pair model, which assignment as a sequential process was challenged due to the co-existing direct and indirect strong-field ionization mechanisms^22,23^. Furthermore, in the case of methanol, Itakura et al. demonstrated the non-linear sensitivity of the dication dissociation spectra and branching ratios to the exact strong-field laser parameters^24,25^. Nevertheless, in a recent pump-probe study implementing consecutive strong-field (~10^14 ^W/cm^2^) laser pulses, Ekanayake et al. reported ${\mathrm{H}}_3^ +$ product signal appearance on an ultrafast (~100 fs) time scale^15^. However, in another recent pump-probe study, also implementing strong-field lasers with similar peak intensities albeit with shorter pulse lengths, Yamanouchi and co-workers were able to observe a ~38 fs beating in the time resolved ${\mathrm{H}}_3^ +$ product signal, which time scale they assigned not to its formation dynamics on the final dication but rather to the vibrations of a singly-ionized intermediate^14^. Evidently, strong-field double-ionization can proceed via different competing direct as well as indirect mechanisms, involving intermediate state dynamics that may mask the time evolution of the making and breaking of chemical bonds^22,23^.

In addition to these experimental challenges, strong-field laser ionization is also very difficult to follow using computational simulations. Most theoretical modeling efforts do not address the strong fields directly. Recent ab initio molecular dynamics (AIMD) simulations on the methanol dication ground state showed ~4% probability for the dominant $${\mathrm{H}}_3^ +$$ formation channel^26^. However, those ground-state dynamics simulations could not account for the experimentally measured branching ratios or predict many of the experimentally observed products such as the C–O bond cleavage or proton migration to form $${\mathrm{H}}_2{\mathrm{O}}^ +$$^15,26^. As we discuss below, these appear only when excited dicationic states and inclusion of non-adiabatic dynamics is allowed.

The purpose of this paper is to develop a combined experimental and theoretical approach that time-resolves the roaming H_2_ chemistry responsible for the H_3_^+^ formation. Taking advantage of high-order harmonic generation (HHG) of ultrafast EUV pulses^16,27,28^, we implement a single-photon double-ionization pump, followed by a time-delayed near-IR (nIR) probe. In this way the new approach not only bypasses the underlying uncertainty in strong-field laser experiments, but is also suitable for analysis using ab initio theoretical modeling^16^. We use XMS-CASPT2/(8e,8o)/aug-cc-pVDZ/potential surfaces and nonadiabatic coupling terms calculated by the BAGEL code^29^, interfaced with a modified version Newton-X (v1.4.0)^30,31^. In our previous work we demonstrated that this level of theory, simulated for the singlet states manifold, allows for a detailed and multifaceted agreement with the Coulomb explosion (CE) product branching ratios, channel-specific kinetic energy release (KER) and three-body momentum correlation spectra, measured in time-independent experiments^28^. Here, the theoretical simulations have paved the way for the experimental timing of the roaming H_2_ dynamics that forms H_3_^+^ on the low lying states of the dication by excitation to higher lying states, which quench H_3_^+^ formation and enhance three-body fragmentation. The combined pump-probe measurements and the nonadiabatic AIMD trajectory simulations allows us to unambiguously conclude that ${\mathrm{H}}_3^ +$ formation in double-ionization of methanol occurs on an ultrafast sub 100 fs time scale, in direct competition with the H_2_^+^ formation channel, settling previous conflicting experimental findings^15,17^.

## Results and discussion

### Methanol dication dynamics

**Fig. 1:**
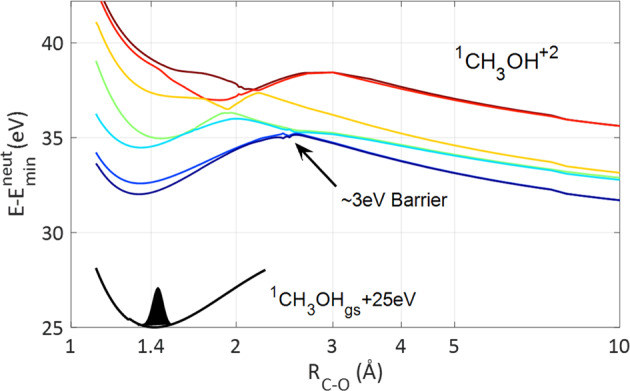
The adiabatic potential curves for the C–O bond breaking channel of CH_3_OH^2+^ using MS-CASPT2/(12e,10o)/aug-ccpVTZ^16^. As the C–O strech coordinate is extended away from the Frank-Condon geometry of the neutral methanol ground state, shown by the black curve, the dication potentials exhibit a ~3 eV barrier that prevents the C–O bond breaking on the low lying states. The potentials are calculated while keeping all other coordinates at their neutral methanol ground state value.

Figure 1 shows the potentials of the low- and the high-lying energy states of the methanol dication as a function of the CO stretch. The dynamics in the latter case typically results in rapid dissociation, accompanied by cleavage of the C–O bond as well as a three-body breakup^16^. In contrast, the dication formed in the low-lying states initially exhibits a potential charge imbalance that hinders direct CE due to the ~3 eV potential barrier indicated in Fig. 1. As a consequence dynamics on the low-lying states are prolonged and highly complex, involving the emergence of a roaming neutral ${\mathrm{H}}_2$.

**Fig. 2:**
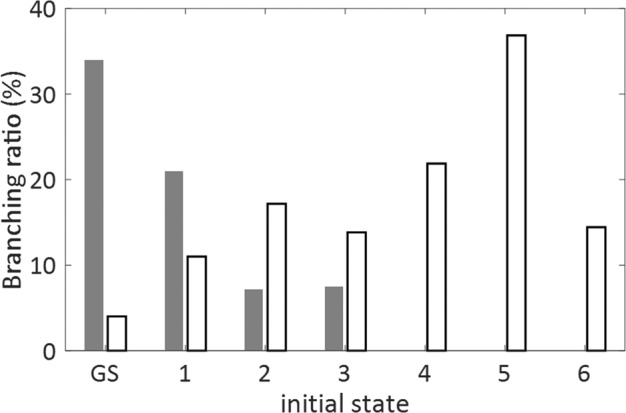
AIMD simulated branching ratio as function of the initial dication state. Full bars represent ${\mathrm{H}}_3^ +$ formation probability and empty bars represent the probability of three-body breakup.

Figure 2 compares the simulated branching ratios for the ${\mathrm{H}}_3^ +$ product of roaming ${\mathrm{H}}_2$, with the combined branching ratio for the three-body breakup channels, calculated for each initial excitation of the methanol dication. Over 1/3 of the ground-state trajectories produce ${\mathrm{H}}_3^ +$, this compared with ~4% obtained in previous ground-state simulations that did not include the second order perturbation theory corrections^26^. The high ${\mathrm{H}}_3^ +$ formation probability on the ground state, drops for the higher lying states and is completely quenched once the CO bond cleavage becomes possible above the third excited state. In contrast, the three-body breakup exhibits an opposite trend, which increases for higher-lying excited states.

These theoretical predictions provide a handle for the experimental time-resolved probing of the dynamics using a delayed nIR pulse, following excitation with the ultrafast EUV pulse. Where the time delayed probe will excite the transient dication to higher-lying states, consequently quenching ${\mathrm{H}}_3^ +$ formation and enhancing three-body breakup. However, once the excess internal energy is released in a successful CE, the product branching is expected to be less affected by the probe pulse.

**Fig. 3:**
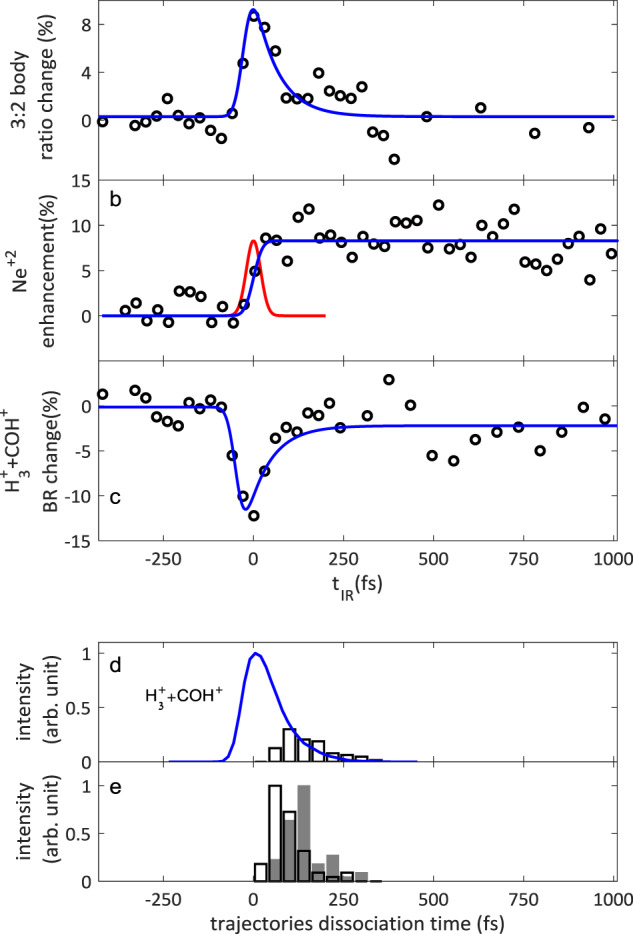
Time resolved experimental and theoretical data. **a** Measured enhancement of three:two body ratio, **b** measured transient $Ne^{2 + }$ cross correlation signal, which rise time is fitted to characterize the instrumental response function, shown by the dotted red line, **c** measured ${\mathrm{H}}_3^ + + \mathrm{COH}^ +$ branching ratio transient depletion. All recorded as function of nIR probe delay with respect to the EUV pump pulse, with statistical error bars on the order of up to 5%. **d** Simulated ${\mathrm{H}}_3^ + + \mathrm{COH}^ +$dissociation times distribution. Blue curve represents the corresponding experimental pump-probe response, convoluted with the measured instrumental response function. **e** Simulated neutral H_2_ separation times distribution and the inverse harpooning times distribution in ${\mathrm{H}}_2^ + + \mathrm{CHOH}^ +$ forming trajectories are shown by the empty and full gray bars respectively.

In designing the probe pulse, we ensure that its peak intensity is kept well below the threshold for strong-field CE, such that at long negative time delays the branching ratios are identical to the ratios measured with the EUV pulse alone. In particular, the ${\mathrm{H}}_3^ +$ formation branching ratio is 6% and the three:two body ratio is 3 to 1. The effect of the nIR probe pulse delay (with respect to the EUV pulse) on the relative enhancement of the three:two body ratio is shown in Fig. 3a. The three:two body ratio increases by up to ~8% at positive time delays, as the nIR probe arrives shortly after the dication formation. This effect decays as the probe pulse arrives and positive time delays longer than ~70 fs. For comparison, Fig. 3b shows the enhancement of doubly-ionized ${\mathrm{Ne}}^{2 + }$ yield as a function of the nIR probe delay that reflects the instrumental response time. The full line in Fig. 3b represents a fit of the ${\mathrm{Ne}}^{2 + }$ yield, assuming photoionization of high lying ${\mathrm{Ne}}^{ + \ast }$ cations by the time delayed nIR pulse, which rise time reflects the cross-correlation of the EUV and nIR pulses. The dashed red line represents the corresponding Gaussian cross-correlation function, in agreement with the <35 fs FWHM of our laser pulses. Figure 3c shows the time correlated relative change in the ${\mathrm{H}}_3^ + + {\mathrm{COH}}^ +$ branching ratio, which exhibits upto ~12% suppression. The full lines in Figs. 3b and 3c show a model trace including an exponential ~70 ± 25 fs lifetime, convoluted with the instrumental time response directly determined based on the ${\mathrm{Ne}}^{2 + }$ data shown in Fig. 3b. While the three:two body ratio appears to return to its unperturbed value, the asymptotic ${\mathrm{H}}_3^ +$ formation remains suppressed by 2.5% even at long time delays. It should be mentioned that while the energy needed to dissociate the ${\mathrm{H}}_3^ +$ ground state is ~4.5 eV^32^, nIR photodissociation of the highly vibrationally excited ${\mathrm{H}}_3^ +$ can still be expected^33,34^. We therefore assign the residual H_3_^+^ depletion at long times to photodissociation of the vibrationally hot ${\mathrm{H}}_3^ +$ cations after the CE is completed.

**Fig. 4:**
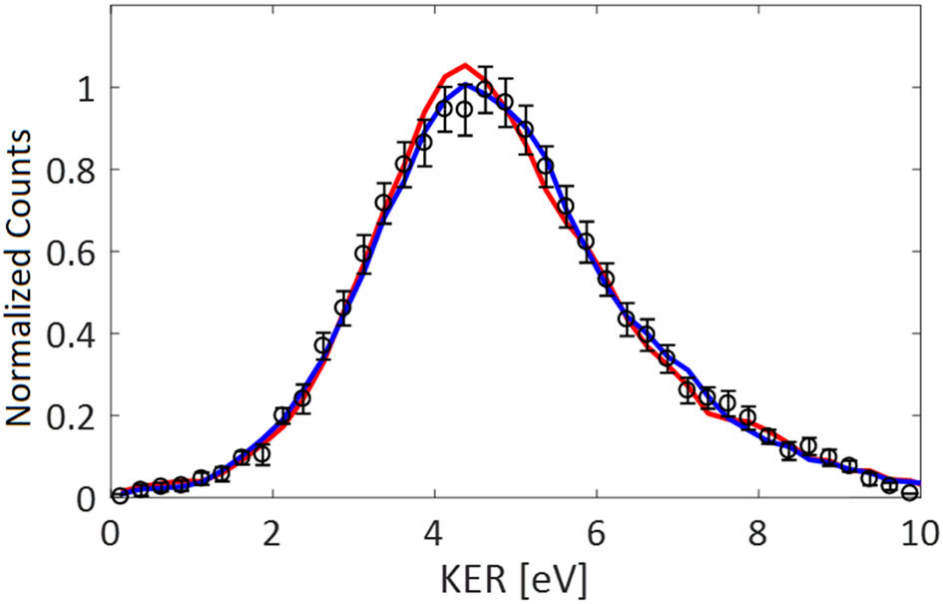
Measured H3^+^+ COH^+^ KER spectra. Comparing spectral shapes for three t_nIR_ windows: *t*_nIR_ < −150fs, −60 fs < *t*_nIR_ < 60 fs and 150 < *t*_nIR_, shown by the solid red line, black circles and blue solid lines respectively.

To provide additional insight into the nIR probe mechanism we compare, in Fig. 4, the shapes of normalized KER distributions, collected at different pump-probe delays. The red line shows the KER measured for negative times, where the nIR probe arrives over 150 fs before the EUV pulse. Similar to the branching ratios, the KER spectrum at negative times is identical to the one measured with the EUV pulse only. The blue line shows the KER at positive delays longer than 150 fs, while the open circles show the KER during the transient suppression times. Thus, while the branching ratios reveal clear time dependence, the KER spectra are not significantly affected by the nIR pulse. We therefore conclude that the field of the nIR probe acts as a switch between H_3_^+^ formation and three-body breakup but does not significantly change transient dynamics leading to the specific channel, as suggested for other experiments implementing EUV pump and a strong-field nIR probe^27,35^. Photo-excitation of the transient time-evolving dication before the CE is expected to promote also competing C–O bond breaking channels and enhances fragmentation as predicted by the AIMD simulations. Interestingly, the C–O bond breaking branching ratio does not exhibit a clear time evolution within the experimental error bars (not shown), possibly due to its competing enhancement on the higher lying states and suppression by three-body fragmentation^28^. For the less abundant CE channels of H^+^ + CH_3_O^+^, H_2_^+^ + CH_2_O^+^ and H_2_O^+^ + CH_2_^+^, statistical errors limit the determination of their individual time evolving branching ratios.

**Fig. 5:**
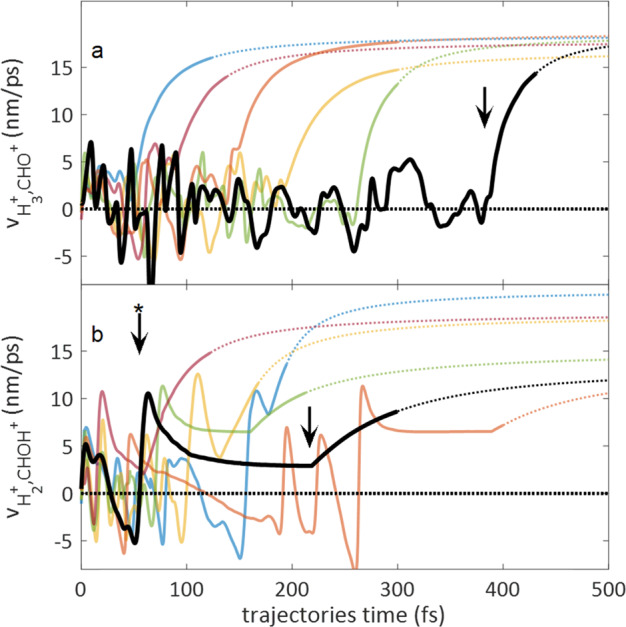
Simulated trajectories. **a** Typical trajectories towards the formation of ${\mathrm{H}}_3^ + + \mathrm{COH}^ +$ showing time evolution of relative velocity of ${\mathrm{H}}_3^ + + \mathrm{COH}^ +$ and **b** towards the formation of ${\mathrm{H}}_2^ + + \mathrm{CHOH}^ +$ via “inverse harpooning” showing time evolution of relative velocity of ${\mathrm{H}}_2^ + + \mathrm{CHOH}^ +$.

Further dynamical insight can be obtained from analysis of the AIMD trajectories culminating in the formation of ${\mathrm{H}}_3^ +$. Figure 5a shows the time evolving inter-fragment velocity, corresponding to the time derivative of the distance between the ${\mathrm{H}}_3^ +$ and ${\mathrm{COH}}^ +$ products. Before dissociation occurs, the inter-fragment velocity exhibits oscillations between positive and negative values, reflecting the roaming ${\mathrm{H}}_2$ motion away from and towards the HCOH^2+^, prior to the formation of ${\mathrm{H}}_3^ +$ as can be seen in the typical AIMD movies provided (see Supplementary Movie 1). Interestingly, Palaudoux et al proposed a possible concerted mechanism for ${\mathrm{H}}_3^ +$ ejection from a ${\mathrm{CH}}_3{\mathrm{Cl}}^{2 + }$ dication^8^. However, none of the trajectories simulated here could be attributed to a concerted ejection of ${\mathrm{H}}_3^ +$. The arrow in Fig. 5a indicates the dissociation time assigned to the highlighted black trajectory, where for each trajectory the dissociation time is defined as the last point of attraction between two dissociating fragments, after which their relative velocity is monotonically increasing until reaching the asymptotic KER. The bars in Fig. 3d show the histogram of the total of 66 trajectories resulting in ${\mathrm{H}}_3^ + + {\mathrm{COH}}^ +$ dissociation, peaking at ~100 fs. While explicit theoretical modeling of the nIR probe is beyond the scope of this paper, the experimental time resolved branching ratios can be compared with the simulated suppression time window that tentatively extends from the formation of each simulated dication until its dissociation time. The full line in Fig. 3d shows the average simulated suppression, constructed from the 66 trajectories that form ${\mathrm{H}}_3^ +$ and convoluted with the experimental instrumental response, in good agreement with the transient branching ratio measurements and an ultrafast sub 100 fs lifetime.

Previous simulations also showed similar roaming H_2_ dynamics within the dication and ultrafast ${\mathrm{H}}_3^ +$ formation^18,26^. However, to understand the ultrafast lifetime of the roaming H_2_ it is important to consider also its other decay channels. Earlier AIMD simulations using CISD and CASSCF electronic potentials reported unbalanced charge dissociation of the methanol dication ground state to form ${\mathrm{H}}_2 + {\mathrm{CHOH}}^{2 + }$, with over 11% and 18% branching ratio respectively^18,26^. In contrast, the non-adiabatic AIMD simulations using CASPT2 potentials indicate that the neutral ${\mathrm{H}}_2$ cannot escape, it is polarized and bound by the ${\mathrm{CHOH}}^{2 + }$ dication. Like the charge-transfer barrier preventing C-O bond cleavage with unbalanced charge, clearly visible in Fig. 1, the neutral ${\mathrm{H}}_2$ cannot dissociate before charge is transferred and CE can proceed. In the ${\mathrm{H}}_3^ +$ formation mechanism the abstraction of a third proton from either methyl site or hydroxyl site allows dissociation by the transfer of a proton. This mechanism is in direct competition with the transfer of an electron that results in a ${\mathrm{H}}_2^ + + {\mathrm{CHOH}}^ +$ breakup. Figure 5b represents typical AIMD trajectories evolving towards ${\mathrm{H}}_2^ +$ formation, showing the inter-fragment velocity between the ${\mathrm{H}}_2^ +$ and ${\mathrm{CHOH}}^ +$ products. The highlighted trajectory exhibits the “inverse harpooning” mechanism^28^, observed in all the trajectories resulting in the ${\mathrm{H}}_2^ + + {\mathrm{CHOH}}^ +$ breakup. The star labeled arrow indicates the time at which a neutral ${\mathrm{H}}_2$ molecule begins to separate from the ${\mathrm{CHOH}}^{2 + }$ dication on the highlighted black trajectory. Although the relative velocity continues to be positive until the asymptotic dissociation limit is reached, the neutral ${\mathrm{H}}_2$ is still bound by the ${\mathrm{CHOH}}^{2 + }$dication. This is evident from the deceleration of the relative velocity. Although reaching long inter-fragment distances, as high as 9 angstroms, no neutral ${\mathrm{H}}_2$ escape. Eventually, at a time indicated by the second arrow, a long-range adiabatic electron transfer from the neutral ${\mathrm{H}}_2$ to the ${\mathrm{CHOH}}^{2 + }$ ignites a CE, producing ${\mathrm{H}}_2^ +$. The charge transfer is evident both from the computed Mulliken charges as well as from the sudden transition to a long acceleration to a high asymptotic KER, typical of the long-range Coulomb repulsion. The open and full bars in Fig. 3e show histograms of the times assigned to the neutral ${\mathrm{H}}_2$ separation and the inverse harpooning times respectively. Interestingly, the competing proton and electron transfer mechanisms that facilitate the release of the two molecular hydrogen ions proceed on comparable ultrafast time scales of ~100 fs, in agreement with the measured ${\mathrm{H}}_3^ +$ suppression time window on the transient dication. This competition can be directly visualized in a selected trajectory (see Supplementary Movie 1), in which the roaming neutral ${\mathrm{H}}_2$ significantly separates from the dication, typical of “inverse harpooning”. Nevertheless, it is pulled back to abstract a proton from the hydroxyl and form ${\mathrm{H}}_3^ +$.

By using a low-field ultrafast EUV pulse to doubly ionize methanol and a time delayed nIR probe pulse it is possible to remove the intrinsic uncertainties of strong-field laser experiments^14,15,26^. A ~70 fs lifetime is observed for the transient suppression of ${\mathrm{H}}_3^ + + {\mathrm{COH}}^ +$ branching ratio, accompanied with a correlated enhancement of the three:two body fragmentation ratio. Furthermore, non-adiabatic AIMD simulations on the ground and excited CASPT2 potentials provide a detailed picture of the ultrafast roaming ${\mathrm{H}}_2$ dynamics, culminating in the trihydrogen product on an ultrafast ~100 fs time scale, in agreement with the experimental result. In contrast to earlier simulations that did not include second-order perturbation theory corrections and predict significant ejection of neutral ${\mathrm{H}}_2$^26^, the simulated roaming neutral ${\mathrm{H}}_2$ was found to exhibit ultrafast competition between proton abstraction resulting in ${\mathrm{H}}_3^ +$ and the “inverse harpooning”, a long-range electron transfer that results in the ${\mathrm{H}}_2^ + + {\mathrm{CHOH}}^ +$ CE channel. Further experimental and theoretical work on deuterated methanol as well as other organic systems will allow to provide a more detailed understanding of the different pathways for ${\mathrm{H}}_3^ +$ formation, explored so far only by strong-field laser experiments. Such ultrafast roaming ${\mathrm{H}}_2$ chemistry, accompanied by competing proton and electron transfer dynamics described here are expected to occur also in other ionized systems produced by ionizing radiation damage, e.g., by cosmic radiation in planetary and interstellar environments or manmade light sources for single molecule crystallography experiments^19,36–38^.

## Methods

### Experimental

The single-photon CE imaging setup has been described earlier^16,27,28^. Briefly, in our experiments, a ~7 mJ, <35 fs, 803 nm near IR (nIR) pulses from a Solstice^39^ laser are split into a 2.1 mJ pump and 4.9 mJ probe pulses. The pump pulse is focused in a semi-infinite neon gas cell, for HHG of ultrafast EUV pulses at a 1 kHz repetition rate^40,41^. Where the EUV is spatially filtered from the higher divergence nIR fundamental^42^. The remaining ~4.9 mJ nIR pulse is time delayed to probe the EUV initiated dynamics and is merged with the ultrafast EUV pulse at a small ~1 degree angle at the center of a home built 3D coincidence imaging spectrometer^16,43^, where both beams cross a skimmed effusive beam of CH_3_OH. The nIR beam is mildly focused behind the spectrometer with a 610 mm lens, ~203 mm behind the spectrometer, such that at the nIR beam is inside the EUV, as validated by using the spectrometer to image the parent ion birth positions. The cationic products are accelerated from the interaction volume towards a time and position sensitive detector, allowing 3D coincidence imaging of the ion recoil velocities^43–45^. Low count rate and center of mass momentum conservation are used to suppress random coincidence cation signal due to dissociative ionization of two different parent molecules. Any residual contributions from random coincidence are estimated and subtracted based on the measured single cation event probabilities. The time resolved Ne^2+^ signal shown in Fig. 3b was used to characterize the temporal overlap of the EUV and nIR pulses, while singly ionized methanol signal was used to monitor the temporal overlap during the long acquisition times. Both 3-body enhancement and ${\mathrm{H}}_3^ +$ depletion were fitted together using the $Ae^{ - \frac{t}{\tau }}\left( {1 + {\mathrm{erf}}\left( {\frac{t}{{\sqrt 2 \sigma }} - \frac{\sigma }{{\sqrt 2 \tau }}} \right)} \right)$ functional form^46^. Where the cross-correlation width $\sigma$ was fixed according to the Ne^2+^ rise time analysis. The free parameters were the respective enhancement\ depletion amplitudes and a common lifetime $\tau$ for both processes. In addition to the transient depletion of ${\mathrm{H}}_3^ +$, a second term with an infinitely long lifetime was added to describe the residual ${\mathrm{H}}_3^ +$ depletion at long time delays.

### Theoretical

The initial configurations (geometries and velocities) of ground state methanol were sampled from a 300 K AIMD simulation of neutral methanol, calculated at the CASSCF level using the MOLCAS package^47^ at the (14e,10o)/aug-ccpVTZ active space/basis-set level. Each of the 100 configurations was used for initiating non-adiabatic molecular dynamics calculations on each one of the seven lowest-lying electronic excited states of the dication. This generated 700 trajectories altogether. The ensuing non-adiabatic dynamics were approximated using surface-hopping molecular dynamics trajectories^48^ generated at the XMSCASPT2/(8e,8o)/aug-cc-pVDZ/density-fitting level using the BAGEL electronic structure package^29^ within the so-called “SS-SR” contraction scheme^49^ used for internally contracted basis functions in CASPT2, where a vertical shift was set to $0.2E_h$. We checked the basis set convergence by comparing aug-cc-pVDZ and aug-cc-pVTZ results for three single-point calculations: (a) double ionization energy at the Franck Condon point; (b) the excitation energy between the dication ground state and its excited states. (c) the barrier height on the dication ground-state. We found that basis set choice led to a very small relative difference of 1% in all cases. For example, the barrier height on the dication state is nearly 3 eV and the difference between the values given by the two basis sets is 0.02 eV. The BAGEL code was interfaced with a modified version Newton-X (v1.4.0) program^31^ for carrying out the surface hopping dynamics^30^. To facilitate the trajectory calculations, the system in adiabatic state n is allowed to hop only to the state m nearest in energy above or below it (i.e. we neglect the non-adiabatic coupling terms $\tau _{nm}$ unless $\left| {n - m} \right| = 1$). We modified the way Newton-X interfaces with the BAGEL code to enable this approximation. The time step for the NA-AIMD trajectories is 0.3 fs. The ab initio dynamics are typically followed until 300 fs or until the inter-fragment velocities are observed to reach an asymptotic monotonic behavior. At this stage, the effect of the residual long-range Coulomb repulsion on the final velocities is taken into account using the classical equations of motion applied to the center of masses of the cationic fragments. Once the asymptotic fragment identity is determined, the inter-fragment velocities are calculated as the time derivative of the distance between the fragment centers of mass.

## Supplementary information

Description of Additional Supplementary Files

Supplementary Movie 1

## Data Availability

The data that support the findings of this study are available from the corresponding author upon reasonable request.

## Code availability

Simulations were performed using open source programs. Modified code files are available from the corresponding authors upon reasonable request.
