# Supplementary material for: Time-resolving the ultrafast H2 roaming chemistry and H3+ formation using extreme-ultraviolet pulses
Source: Commun Chem. 2020 Apr 21;3:49. doi: 10.1038/s42004-020-0294-1 (PMC9814522; doi:10.1038/s42004-020-0294-1)
Supplement: Supplementary file 1 — Description of Additional Supplementary Files [file 42004_2020_294_MOESM1_ESM.pdf]

Description of Additional Supplementary Files

File Name: Supplementary Movie 1
Description: Ab-initio molecular dynamics movie shows typical H_2 roaming dynamics. Three selected simulations show the mechanisms described in the manuscript (1) H_3^+ formation by proton transfer, (2) H_2^+ formation by e- transfer, and (3) Competition of H_2^+ and H_3^+ formation.
